# *Lactobacillus Reuteri* 6475 Prevents Bone Loss in a Clinically Relevant Oral Model of Glucocorticoid‐Induced Osteoporosis in Male CD‐1 Mice

**DOI:** 10.1002/jbm4.10805

**Published:** 2023-08-13

**Authors:** Nicholas J Chargo, Jonathan D Schepper, Naoimy Rios‐Arce, Ho Jun Kang, Joseph D Gardinier, Narayanan Parameswaran, Laura R McCabe

**Affiliations:** ^1^ Department of Physiology Michigan State University East Lansing MI USA; ^2^ College of Osteopathic Medicine Michigan State University East Lansing MI USA; ^3^ Bone and Joint Center Henry Ford Health System Detroit MI USA; ^4^ College of Human Medicine Michigan State University East Lansing MI USA

**Keywords:** BARRIER, GLUCOCORTICOID, GUT‐BONE INTERACTIONS, OSTEOPOROSIS, PROBIOTIC

## Abstract

Glucocorticoids (GCs) are commonly used anti‐inflammatory medications with significant side effects, including glucocorticoid‐induced osteoporosis (GIO). We have previously demonstrated that chronic subcutaneous GC treatment in mice leads to gut barrier dysfunction and trabecular bone loss. We further showed that treating with probiotics or barrier enhancers improves gut barrier function and prevents GIO. The overall goal of this study was to test if probiotics could prevent GC‐induced gut barrier dysfunction and bone loss in a clinically relevant oral‐GC model of GIO. Eight‐week‐old male CD‐1 mice were treated with vehicle or corticosterone in the drinking water for 4 weeks and administered probiotics *Lactobacillus reuteri* ATCC 6475 (LR 6475) or VSL#3 thrice weekly via oral gavage. As expected, GC treatment led to significant gut barrier dysfunction (assessed by measuring serum endotoxin levels) and bone loss after 4 weeks. Serum endotoxin levels significantly and negatively correlated with bone volume. Importantly, LR 6475 treatment effectively prevented both GC‐induced increase in serum endotoxin and trabecular bone loss. VSL#3 had intermediate results, not differing from either control or GC‐treated animals. GC‐induced reductions in femur length, cortical thickness, and cortical area were not affected by probiotic treatment. Taken together, these results are the first to demonstrate that LR 6475 effectively prevents the detrimental effects of GC treatment on gut barrier, which correlates with enhanced trabecular bone health in an oral mouse model of GIO. © 2023 The Authors. *JBMR Plus* published by Wiley Periodicals LLC on behalf of American Society for Bone and Mineral Research.

## Graphical abstract

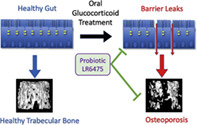

## Introduction

Glucocorticoids (GC) are powerful anti‐inflammatory drugs. Recent estimates suggest that >1% (>3 million, data.census.gov) of the US population is currently undergoing chronic GC therapy to treat a plethora of inflammatory conditions, including asthma, rheumatoid arthritis, and inflammatory bowel disease, among others.^(^ ^1^ ^)^ Although GCs successfully reduce inflammation and provide symptomatic relief, prolonged use often leads to serious side effects, including glucocorticoid‐induced osteoporosis (GIO).^(^ ^2^, ^3^ ^)^ GIO is the leading cause of secondary osteoporosis (a condition caused by another disease or treatment of another disease) and has been well documented in both the basic science and clinical literature.^(^ ^4^, ^5^, ^6^ ^)^ GIO significantly increases the risk of osteoporosis‐related fractures in chronic GC users and leads to increased morbidity and mortality.^(^ ^7^, ^8^, ^9^ ^)^

Current therapeutic options to treat GIO include bisphosphonates, denosumab, and intermittent parathyroid hormone (iPTH), among others.^(^ ^5^ ^)^ These therapeutics, although effective, can have unwanted side effects. For example, the leading cause of bisphosphonate discontinuation is gastrointestinal upset.^(^ ^10^ ^)^ Also, there is a rare yet serious side effect of jaw osteonecrosis with prolonged bisphosphonate use,^(^ ^11^ ^)^ and past guidelines limited lifetime iPTH use to 24 months, although this limit has recently been removed for patients with high fracture risk.^(^ ^12^ ^)^ Given the realized and potential side effects of these treatment options, adherence to treatment is often poor, leading to a high burden of disease and poor patient outcomes (ie, increased fracture incidence). In addition, although GC cessation and use of another anti‐inflammatory drug would be preferred to prevent GIO, this is not an option for some patients. Thus, there is a need to develop novel therapeutic options with fewer side effects that can mitigate GIO and ultimately reduce fracture risk.

Our lab and others have identified the gut microbiota and gut barrier function as important targets in the treatment of many diseases, including several forms of osteoporosis.^(^ ^13^, ^14^, ^15^, ^16^, ^17^, ^18^, ^19^ ^)^ This has led to increased attention on probiotics, bacteria that are beneficial to the health of the host (oxfordreference.com), including gut and bone health in mouse disease models.^(^ ^16^, ^17^, ^20^, ^21^, ^22^, ^23^, ^24^, ^25^, ^26^, ^27^, ^28^ ^)^ Our lab recently demonstrated the beneficial effects of probiotics in preventing GIO in adult male C57BL/6J mice.^(^ ^16^ ^)^ We found that GC treatment alone induced significant trabecular bone loss (a 50% reduction in trabecular bone volume [BV/TV]) that was linked to impaired gut barrier function (leaky gut), whereas treatment with *Lactobacillus reuteri* ATCC 6475 (LR 6475) maintained gut barrier function and completely prevented the GC‐induced trabecular bone loss.^(^ ^16^ ^)^ The bone health benefits of *L. reuteri* have also been reported clinically where treatment slows bone loss in elderly women.^(^ ^29^ ^)^ Together, these studies clearly demonstrate an important role for probiotics, particularly LR 6475, and barrier function in the treatment of osteoporosis induced by disease or subcutaneous GC administration.

Although the subcutaneous pellet model of GIO is widely utilized in mice and mimics the human bone response, in humans GCs are typically administered orally. Previous studies have reported an oral GC treatment mouse model that develops GIO.^(^ ^30^ ^)^ Specifically, dual‐energy X‐ray absorptiometry measurements identified a reduction in whole‐body bone mineral content and bone area in 8‐week‐old male CD‐1 mice receiving oral GC treatment for 4 weeks.^(^ ^30^ ^)^ Consistent with this finding, a reduction a serum osteocalcin was also reported.^(^ ^30^ ^)^ A benefit of studying CD‐1 mice is that they are an outbred strain and have more genetic variability, which makes observed responses less likely to be the result of specific clonal genetics and more relevant to the human population. In this model, since 8‐week‐old mice are skeletally immature and still growing, there is also relevance to growing children who receive chronic GC treatment for various medical conditions (inflammatory diseases, congenital adrenal hyperplasia, genetic conditions, etc.).

Therefore, we tested if LR 6475 can prevent bone loss in an oral GC mouse model (CD‐1 strain) via administration of GC in the drinking water. Consistent with our previous studies in a different model, we demonstrate here that LR 6475 prevents oral GC‐induced gut barrier dysfunction and trabecular bone loss, indicating that oral GC delivery does not attenuate the beneficial effect of the probiotic on GC‐induced bone loss. This has important translational implications on the use of probiotic LR 6475 in human osteoporosis.

## Materials and Methods

### Animals and experimental design

All animal procedures were approved by Michigan State University Institutional Animal Care and Use Committee and conformed to NIH guidelines. Seven‐week‐old male CD‐1 Swiss white mice (*n* = 32, 8/group) were obtained from Charles River Laboratories (Wilmington, MA, USA) and were allowed to acclimate to the facility for 1 week before beginning experiments. Male mice were used in this study to mimic and test the original oral corticosterone model utilized by Gasparini and colleagues, which was performed in CD‐1 male mice.^(^ ^30^ ^)^ An advantage of using male mice in this study is that they have greater bone mass than females, allowing us to readily detect bone loss in response to glucocorticoid treatments. Future studies will include female mice to assess the preclinical efficacy of probiotics in both sexes. Animals were housed at 4 mice per cage, on a 12‐hour light/dark cycle and had *ad libitum* access to sterilized standard chow (Teklad 2019, Teklad, Madison, WI, USA) and water. Upon reaching 8 weeks of age, each cage was randomly assigned to a treatment group and mice were treated with corticosterone (Sigma C2505, Sigma‐Aldrich, St. Louis, MO, USA) for 4 weeks. Corticosterone (CS) was dissolved in 100% ethanol and added to the drinking water to a final concentration of 75 μg CS/mL (~9 mg/kg/d) and 0.4% ethanol. Vehicle controls were treated with water containing 0.4% ethanol. Water was changed weekly to maintain freshness. Animals were also treated with probiotics *Lactobacillus reuteri* ATCC 6475 (LR 6475) or VSL#3 (Alfasigma, Covington, LA, USA). VSL#3 is a multi‐strain probiotic containing *Streptococcus thermophilus*, *Bifidobacterium breve*, *Bifidobacterium lactis* (previously *B. longum and B. infantis*), *Lactobacillus acidophilus*, *Lactobacillus plantarum*, *Lactobacillus helveticus*, and *Lactobacillus paracasei*. Three hundred microliters of probiotic was gavaged three times per week at midday throughout the duration of the 4‐week treatment at a dose of 1 × 10^9^ CFU/mL. Control animals were gavaged with 300 μL of culture broth lacking bacteria.

### Probiotic culture/preparation

LR 6475 was cultured under anaerobic conditions on de Man, Rogosa, and Sharpe media (MRS; DIFCO, Becton Dickinson, Franklin Lakes, NJ, USA) plates. Plates were kept at 37°C for a maximum of 1 week. For gavage, single bacterial colonies were cultured in 10 mL of MRS broth. After 16 to 18 hours at 37°C, animals were gavaged with 300 μL of bacteria (1 × 10^9^ CFU/mL). VSL#3 capsules (112.5 billion CFU/capsule) were dissolved in sterile deionized water to a final concentration of 1 × 10^9^ CFU/mL, and animals were gavaged with 300 μL.

### Microcomputed tomography (μCT) bone analysis

Femurs and vertebrae collected during harvest were scanned in a GE Explore Locus μCT (GE Healthcare, Piscataway, NJ, USA) at a resolution of 20 μm obtained from 720 views. Each scan had bones from each treatment group and was phantom calibrated to maintain consistency. A fixed threshold of 1000 was used for analysis on all bones. The distal femur trabecular bone region was defined as 10% proximal to the distal growth plate based on total bone length and excluded cortical bone. Trabecular bone was also analyzed within the body of the L_4_ vertebrae. Trabecular bone parameter values, including volume, thickness, spacing, and number, were obtained using GE Healthcare MicroView software version 2.2. Femoral cortical bone was analyzed within a 2 × 2 × 2 mm cube centered in the midshaft of the femur. All bones were analyzed blinded to treatment group.

### Serum measurements

Sterile blood was collected via cardiac puncture during harvest and allowed to clot at room temperature for 5 minutes before being temporarily stored on ice. Blood was centrifuged at 10,000*g* for 10 minutes and serum was removed, aliquoted, and snap‐frozen in liquid nitrogen and stored at −80°C until further use. Serum bacterial endotoxin levels were determined using the HEK‐Blue LPS Detection Kit (InvivoGen, San Diego, CA, USA) according to manufacturer's protocol. Serum samples went through no more than two freeze/thaw cycles to ensure integrity.

### Biomechanical testing

Mechanical properties were measured in the femur under three‐point bending using an EnduraTech ELF 3200 Series (Bose, Framingham, MA, USA). The base support span was 6 mm wide with the loading point positioned in the middle. The femur was positioned on the base support so that the posterior surface was under tension. The femur was then loaded at a rate of 0.025 mm/s until fracture, while force and displacement were recorded. The force‐displacement curve was used to determine structural‐level mechanical properties. Analyses were done blind to the experimental condition of the sample.

### Statistical analyses

All analyses were completed blinded to treatment group. Violin plots show the range of values with lines at the median and quartiles. Values in tables are presented as mean ± SEM. No outliers were removed; however, one mouse from the GC + LR 6475 group was removed from all bone analyses because of improper bone preservation at the time of harvest. Analysis was performed via one‐way ANOVA with Tukey's post‐test using GraphPad Prism software version 9 (GraphPad, San Diego, CA, USA). If standard deviations were significantly different via Bartlett's test, one‐way ANOVA was performed with Brown–Forsythe and Welch ANOVA tests with Dunnett's T3 post‐test.

## Results

### Body weights and femur length

**Fig. 1:**
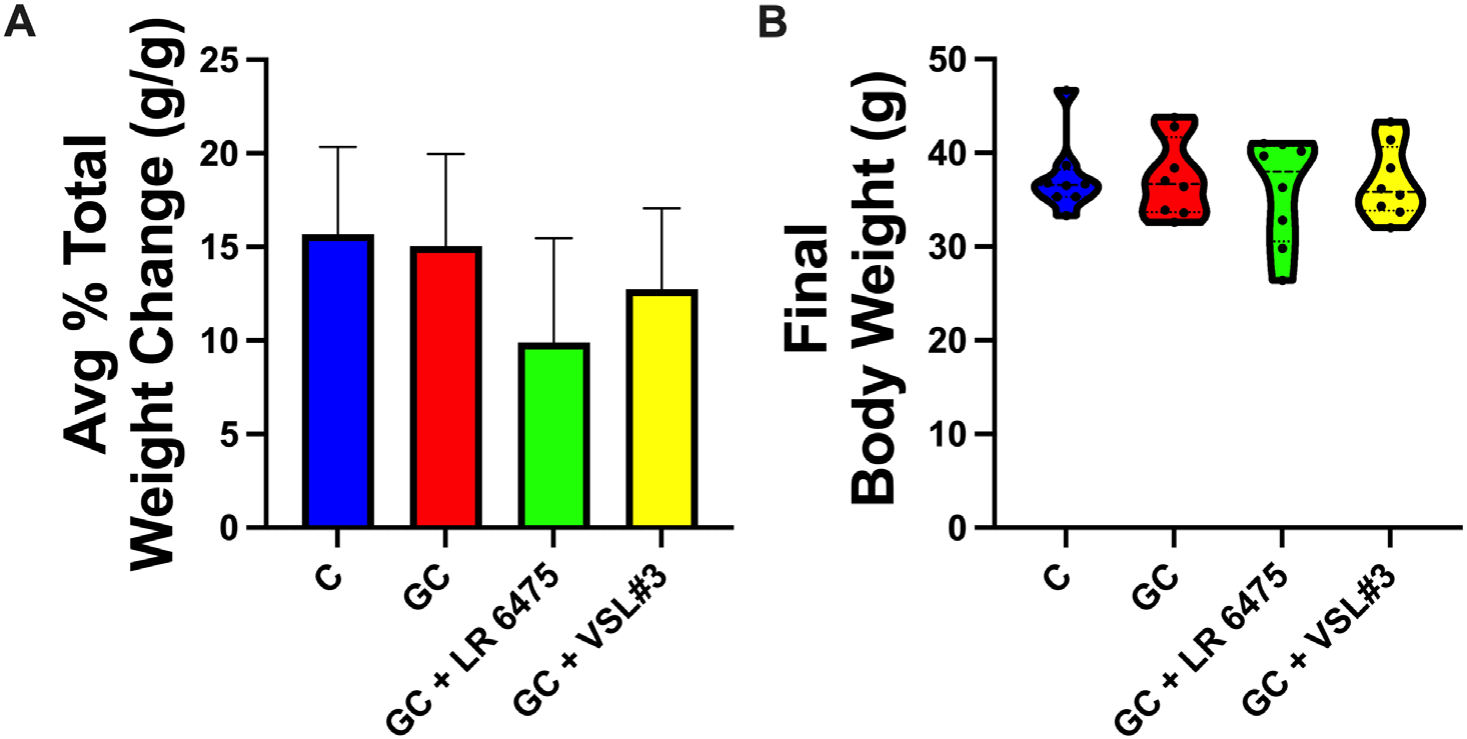
Glucocorticoid and probiotic treatment does not cause differences in body weight. (*A*) Average percent body weight change after 4 weeks of treatment. Bar graph is mean + SEM. (*B*) Final body weights after 4 weeks of treatment. Violin plot represents minimum to maximum values with lines at the median and quartiles. No significant differences were observed. Statistical analyses via one‐way ANOVA with Tukey's post‐test. *n* = 8/group. GC = glucocorticoid; LR 6475 = *Lactobacillus reuteri* ATCC 6475.

Glucocorticoid and probiotic treatment were begun in 8‐week‐old male CD‐1 mice and were continued for 4 weeks. All groups gained a similar amount of body weight, and there were no significant differences at the beginning or end of the 4‐week study (Fig. 1, Table 1). In contrast, 4 weeks of GC treatment resulted in a significant reduction in both spleen weight (*p* < 0.05) and femur length (*p* < 0.0001) in the GC‐treated groups compared with control (Table 1). Probiotic supplementation did not prevent the reduction caused by GC, and all GC‐treated groups remained significantly different from controls (Table 1).

**Table 1:** General Body Parameters Before and After 4 Weeks of Treatment

	Controls	GC	GC + LR 6475	GC + VSL#3
	(*n =* 8)	(*n =* 8)	(*n =* 7–8)	(*n =* 8)
General parameters				
Initial body weight (g)	32.44 ± 0.81	32.49 ± 0.50	32.61 ± 0.44	32.74 ± 0.60
Final body weight (g)	36.09 ± 0.63	37.31 ± 1.48	35.89 ± 1.99	36.85 ± 1.38
Spleen weight (g)	0.088 ± 0.01	**0.048 ± 0.01** ^a^	**0.054 ± 0.01** ^a^	**0.050 ± 0.01** ^a^
Femur length (mm)	16.37 ± 0.06	**15.36 ± 0.17** ^b^	**15.21 ± 0.16** ^b^	**15.08 ± 0.14** ^b^

*Note*: Values reported as mean ± SEM. Statistical analysis performed via one‐way ANOVA with Tukey's post‐test. Bold values from experimental groups denote significant differences from control.

Abbreviations: GC = glucocorticoid; LR 6475 = *Lactobacillus reuteri* ATCC 6475.

^a^ *p* < 0.05.

^b^ *p* < 0.0001 for significant comparisons.

### Bone microarchitectural and cortical analyses

**Fig. 2:**
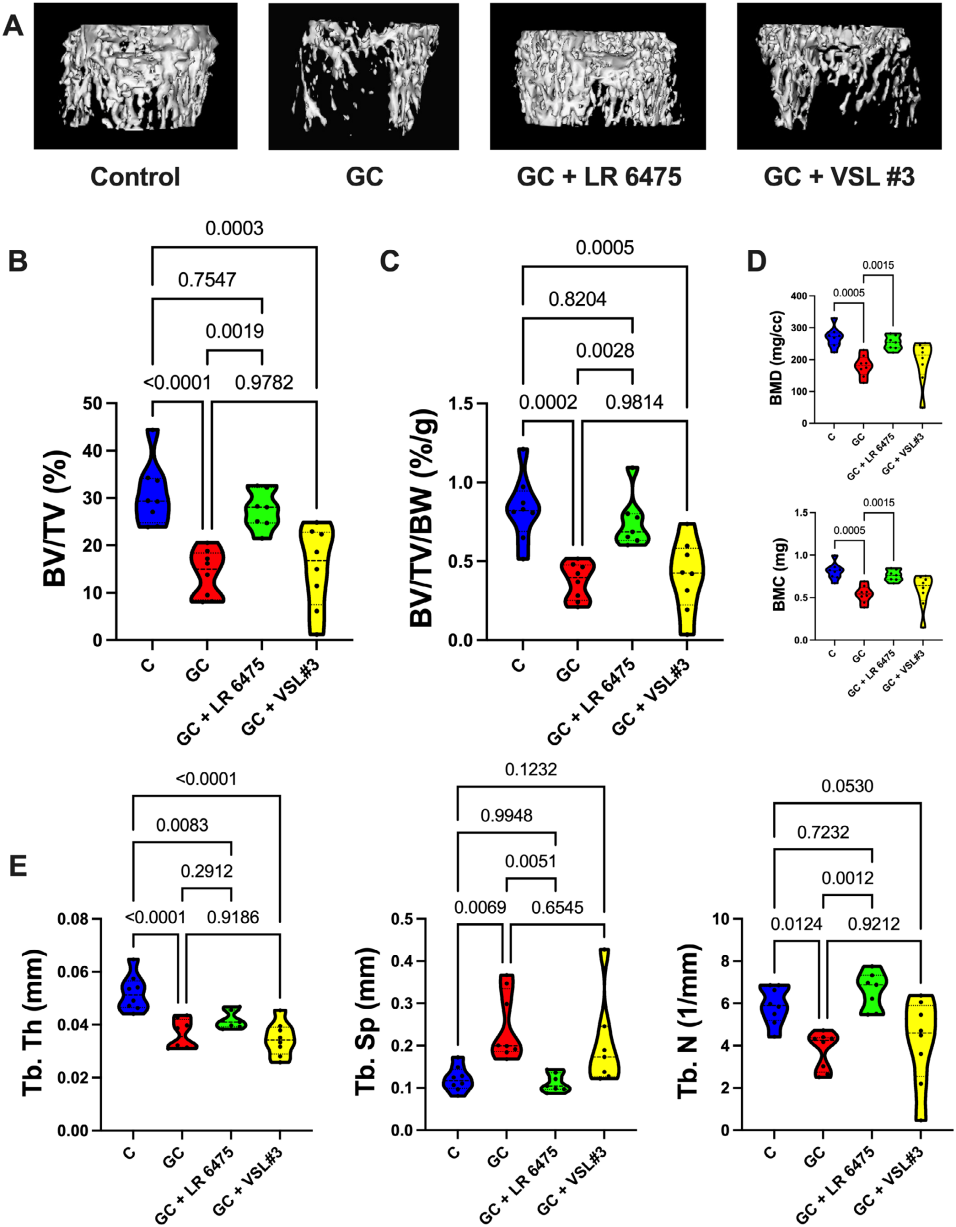
*Lactobacillus reuteri* ATCC 6475 prevents glucocorticoid‐induced distal femoral trabecular bone loss. Eight‐week‐old male CD‐1 mice were treated +/− GC and probiotics LR 6475 or VSL#3 for 4 weeks. (*A*) Representative iso‐surface images of distal femur trabecular bone. (*B*, *C*) μCT analysis of distal femur trabecular bone expressed as (*B*) bone volume fraction and (*C*) bone volume fraction corrected for body weight. (*D*) Distal femur trabecular bone mineral density and bone mineral content. (*E*) Distal femur trabecular bone microarchitectural analyses. *n* = 7–8 per group. Violin plots represent minimum to maximum values with lines at the median and quartiles. Statistical analysis performed via one‐way ANOVA with Tukey's post‐test. BMC = bone mineral content; BMD = bone mineral density; BV/TV = bone volume/total volume; BV/TV/BW = bone volume/total volume/body weight; GC = glucocorticoid; LR 6475 = *Lactobacillus reuteri* ATCC 6475; Tb.N = trabecular number; Tb.Sp = trabecular spacing; Tb.Th = trabecular thickness.

As expected, 4‐week oral GC treatment significantly reduced distal femur metaphyseal bone volume/total volume (BV/TV%) compared with control (control = 30.76 ± 2.38, GC = 14.05 ± 1.74, *p* < 0.0001) (Fig. 2*B*). Oral supplementation with probiotic *Lactobacillus reuteri* ATCC 6475 (LR 6475) prevented this loss, with the GC + LR 6475 group remaining near the control group value (GC + LR 6475 = 27.49 ± 1.54, *p* = 0.7547 compared with control). Supplementing with VSL#3 did not have this bone‐protective effect, with BV/TV% remaining significantly lower than control (*p* = 0.0003) (Fig. 2*B*). To ensure that variation in body weight was not impacting trabecular bone density, distal femur metaphyseal BV/TV% was corrected for body weight and we observed the same results (Fig. 2*C*). Correspondingly, LR 6475 prevented reductions in distal femoral bone mineral density (BMD) and bone mineral content (BMC) induced by GC treatment (Fig. 2*D*). Examining the microarchitectural properties of the distal femur further (Fig. 2*E*), trabecular thickness (Tb.Th) was significantly reduced in all GC‐treated groups (*p* < 0.01 compared with control), and neither probiotic treatment was able to prevent this decrease. Trabecular spacing (Tb.Sp) was significantly increased with GC treatment compared with controls (*p* = 0.0069), and trabecular number (Tb.N) was significantly decreased with GC treatment compared with controls (*p* = 0.0124). Supplementation with LR 6475 completely prevented these changes, whereas VSL#3 did not have a protective effect (Fig. 2*E*). Together, these findings suggest that the bone‐protective effects of probiotics in this oral GIO model may be probiotic specific, with not all probiotics being effective at preventing distal femur metaphyseal bone loss.

Examining cortical bone parameters from the mid‐diaphyseal region of the femur, cortical thickness (Ct.Th) and cortical area (Ct.Ar) were significantly decreased by the 4‐week GC treatment and neither probiotic supplement was able to prevent this loss (*p* < 0.01, Table 2). Additionally, BMC and bone area were significantly reduced in all GC‐treated groups and neither probiotic was able to prevent this loss (*p* ≤ 0.05, Table 2). No significant changes were observed in marrow area, total area, endocortical perimeter, periosteal perimeter, moment of inertia, or bone mineral density. These findings suggest that the mechanism of probiotics' protective effect on bone health may be different in cortical bone compared with trabecular bone. It is also possible that the lack of protective effect in cortical bone may simply be due to the relatively short duration of the study.

**Table 2:** Femur Mid‐diaphyseal Cortical Parameters

	Controls	GC	GC + LR 6475	GC + VSL#3
	(*n =* 8)	(*n =* 8)	(*n =* 7)	(*n =* 8)
Femur cortical parameters				
Ct.Th. (mm)	0.34 ± 0.01	**0.29 ± 0.01** ^b^	**0.29 ± 0.01** ^b^	**0.28 ± 0.01** ^b^
Ct.Ar. (mm^2^)	1.44 ± 0.05	**1.21 ± 0.05** ^b^	**1.21 ± 0.03** ^b^	**1.19 ± 0.03** ^b^
Ma.Ar. (mm^2^)	0.85 ± 0.03	0.87 ± 0.03	0.89 ± 0.06	0.92 ± 0.03
Tt.Ar. (mm^2^)	2.28 ± 0.06	2.08 ± 0.06	2.10 ± 0.08	2.11 ± 0.02
Ec.Pm. (mm)	3.44 ± 0.05	3.49 ± 0.05	3.55 ± 0.13	3.57 ± 0.07
Ps.Pm. (mm)	5.52 ± 0.07	5.30 ± 0.08	5.38 ± 0.16	5.32 ± 0.02
MOI (mm^4^)	0.42 ± 0.03	0.32 ± 0.03	0.39 ± 0.03	0.33 ± 0.03
BMD (mg/cc)	868.5 ± 17.28	839.7 ± 23.10	816.2 ± 12.81	846.2 ± 19.98
BMC (mg)	0.025 ± 0.001	**0.021 ± 0.001** ^a^	**0.020 ± 0.001** ^a^	**0.020 ± 0.001** ^a^
Bone area (mm^2^)	0.629 ± 0.01	**0.579 ± 0.01** ^c^	**0.577 ± 0.02** ^a^	**0.564 ± 0.02** ^b^

*Note*: All values obtained from μCT analysis. Values reported as mean ± SEM. Statistical analyses performed via one‐way ANOVA with Tukey's post‐test. Bold values from experimental groups denote significant difference from control.

Abbreviations: BMC = bone mineral content; BMD = bone mineral density; bone area = cortical area/total area; Ct.Ar. = cortical area; Ct.Th. = cortical thickness; Ec.Pm. = endocortical perimeter; GC = glucocorticoid; LR 6475 = *Lactobacillus reuteri* ATCC 6475; Ma.Ar. = marrow area; MOI = moment of inertia; Ps.Pm. = periosteal perimeter; Tt.Ar. = total area.

^a^ *p* < 0.05.

^b^ *p* < 0.01.

^c^ *p* = 0.0529 for significant comparisons.

**Fig. 3:**
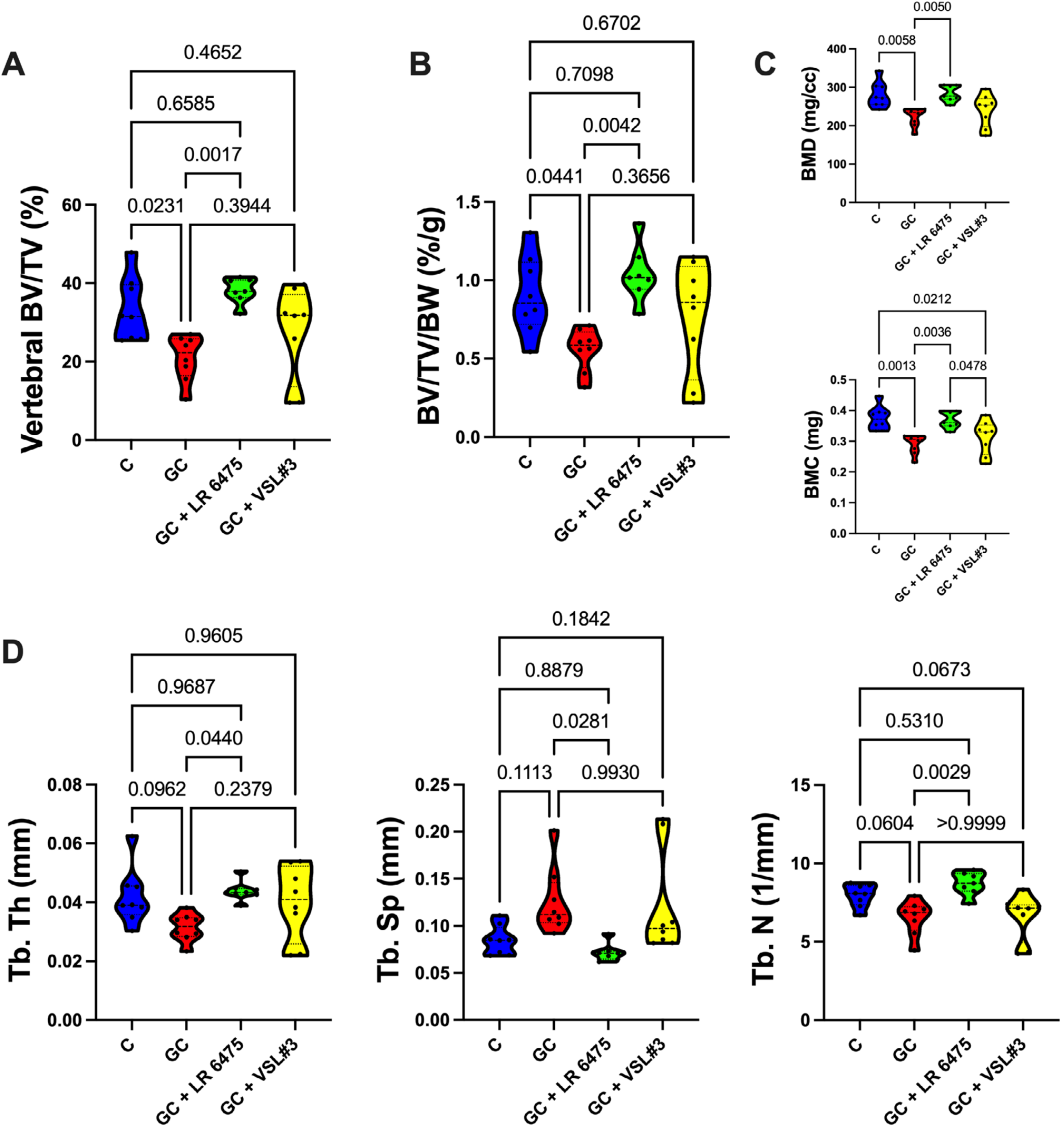
*Lactobacillus reuteri* ATCC 6475 prevents glucocorticoid‐induced vertebral body trabecular bone loss. (*A*, *B*) μCT analysis of vertebral body trabecular bone expressed as (*A*) bone volume fraction and (*B*) bone volume fraction corrected for body weight. (*C*) Vertebral body trabecular bone mineral density and bone mineral content. (*D*) Vertebral body trabecular bone microarchitectural analyses. *n* = 7–8 per group. Violin plots represent minimum to maximum values with lines at the median and quartiles. Statistical analysis performed via one‐way ANOVA with Tukey's post‐test. BMC = bone mineral content; BMD = bone mineral density; BV/TV = bone volume/total volume; BV/TV/BW = bone volume/total volume/body weight; GC = glucocorticoid; LR 6475 = *Lactobacillus reuteri* ATCC 6475; Tb.N = trabecular number; Tb.Sp = trabecular spacing; Tb.Th = trabecular thickness.

To assess an axial bone site, we examined the trabecular bone of the L_4_ vertebral body. We observed a significant reduction in trabecular BV/TV% in the vertebrae with GC treatment compared with control (*p* = 0.0231) and LR 6475 prevented this decrease (*p* = 0.0017 compared with GC). Responses to VSL#3 treatment were variable, and the VSL#3‐treated group was not statistically different from control or GC‐treated groups (Fig. 3*A*). These results are the same when corrected for body weight (Fig. 3*B*). Similarly, when looking at BMD and BMC, LR6475 prevented GC reductions in BMC and BMD (*p* < 0.01, Fig. 3*C*). When looking more closely at the trabecular microarchitecture (Fig. 3*D*), no differences were observed compared with control; however, LR 6475 was significantly different from GC alone in all three measures (Tb.Th, Tb.Sp, and Tb.N, *p* < 0.05). These changes in microarchitecture were more variable with VSL#3 treatment. Taken together, these findings suggest that the effectiveness of probiotics in the vertebrae again may be probiotic specific, with LR 6475 being protective and VSL#3 having a variable effect.

### Femoral biomechanical properties

**Fig. 4:**
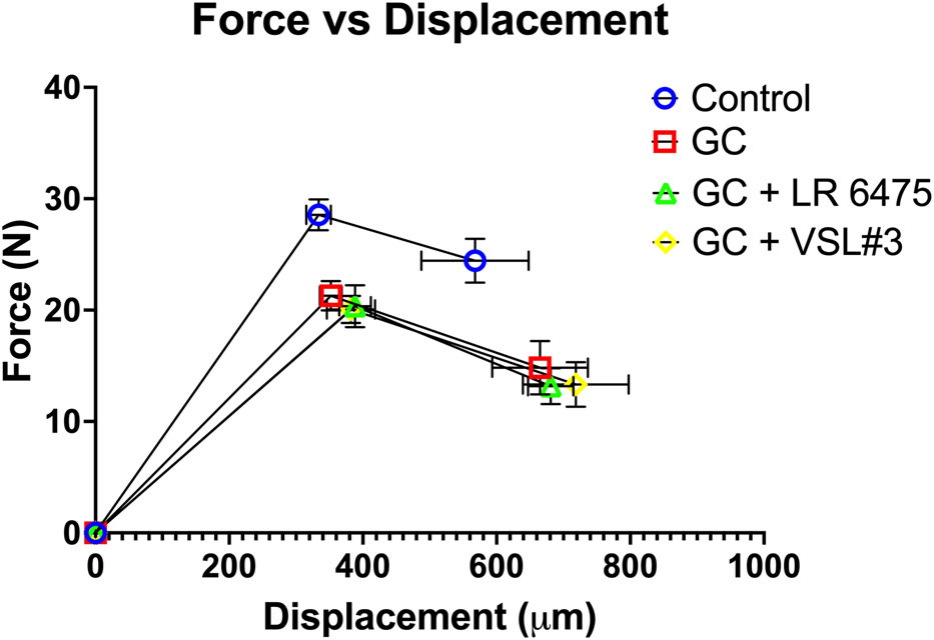
Probiotics do not prevent reduced femur strength caused by glucocorticoid treatment. Data points represent ultimate load and fail load along with respective displacements via three‐point bending to determine structural‐level biomechanical properties. *n* = 6–8. Values are mean ± SEM. Error bars correspond to respective axis. GC = glucocorticoid; LR 6475 = *Lactobacillus reuteri* ATCC 6475.

Three‐point bending of the femur revealed a significant reduction in both ultimate load and fail load in all GC‐treated groups (*p* < 0.05 compared with control; Fig. 4, Table 3), and probiotic supplementation did not have a protective effect. No significant differences were observed in ultimate displacement or fail displacement. Stiffness, a structural‐level biomechanical property of the femur, was also reduced in all GC‐treated groups, and again, probiotics did not have a protective effect (Table 3). These findings demonstrate that GC treatment causes impairments in structural‐level biomechanical properties of the femur and that 4‐week treatment with probiotics does not have a protective role in this model.

**Table 3:** Femur Biomechanical Properties

	Control	GC	GC + LR 6475	GC + VSL#3
	(*n =* 7)	(*n =* 8)	(*n =* 6)	(*n =* 8)
Femur biomechanical properties				
Ult load (N)	28.55 ± 1.51	**21.29 ± 1.41** ^a^	**20.35 ± 2.06** ^b^	**20.07 ± 1.31** ^b^
Fail load (N)	24.44 ± 2.14	**14.83 ± 2.56** ^a^	**13.17 ± 1.76** ^a^	**13.32 ± 2.14** ^b^
Ult disp. (μm)	333.7 ± 19.9	352.0 ± 17.4	388.3 ± 25.8	382.1 ± 38.5
Fail disp. (μm)	567.7 ± 86.7	665.0 ± 76.6	680.8 ± 37.1	718.5 ± 85.6
Stiffness (N/mm)	188.1 ± 12.4	**129.2 ± 6.42** ^b^	**141.8 ± 13.4** ^a^	**131.3 ± 9.27** ^b^
Work (mJ)	11.94 ± 1.63	9.50 ± 0.89	10.44 ± 0.93	10.47 ± 1.38

*Note*: Structural‐level biomechanical properties of femurs were obtained from three‐point bending testing. Values reported as mean ± SEM. Statistical analysis performed via one‐way ANOVA with Tukey's post‐test. Bold values from experimental groups denote significant differences from control.

Abbreviations: Fail disp. = fail displacement; GC = glucocorticoid; LR 6475 = *Lactobacillus reuteri* ATCC 6475; Ult disp. = ultimate displacement; Ult load = ultimate load.

^a^ *p* < 0.05.

^b^ *p* < 0.01.

### Gut barrier function and correlation to bone health

**Fig. 5:**
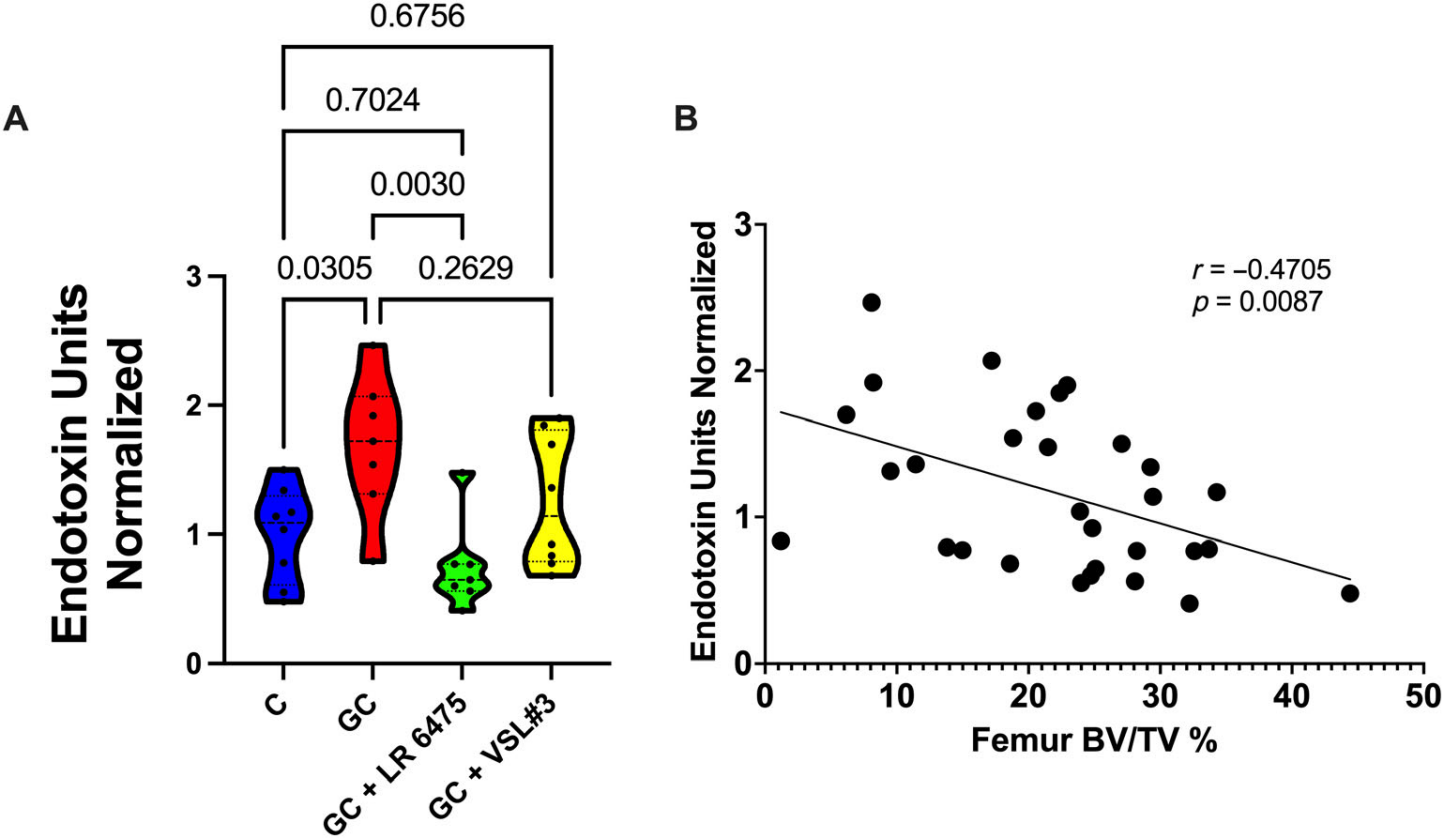
*Lactobacillus reuteri* ATCC 6475 improves gut barrier function. (*A*) Serum endotoxin unit quantification normalized to control levels. (*B*) Correlation analysis between normalized serum endotoxin units and distal femur trabecular BV/TV%. *n* = 7–8 per group. Violin plots represent minimum to maximum values with lines at the median and quartiles. Statistical analysis performed via one‐way ANOVA with Tukey's post‐test and Pearson's correlation analysis. BV/TV = bone volume/total volume; GC = glucocorticoid; LR 6475 = *Lactobacillus reuteri* ATCC 6475.

Gut barrier integrity can be measured by assessing certain gut bacterial components in the serum that are usually absent when the gut barrier is intact. One such component, endotoxin (lipopolysaccharide [LPS]) is present on the outer wall of gram‐negative bacteria. When there is gut barrier dysfunction, gut bacteria and bacterial components including endotoxin can traverse the gut wall, enter the systemic circulation, and be measured in the serum. Thus, an increase in serum endotoxin is indicative of impaired gut barrier integrity (ie, leaky gut). In the present study, normalized serum endotoxin was significantly elevated in GC‐treated compared with control mice (control = 1.00 ± 0.13, GC = 1.689 ± 0.21, *p* = 0.0305). Supplementation with LR 6475 completely prevented this increase. However, response to VSL#3 was more variable (Fig. 5*A*). This finding suggests that LR 6475 supplementation helps maintain the integrity of the gut barrier. In this study, we further observed that relative levels of serum endotoxin negatively correlate with distal femur metaphyseal BV/TV% (*r* = −0.4705, *p* = 0.0087; Fig. 5*B*), similar to our previous reports.^(^ ^16^ ^)^ Serum endotoxin levels did not correlate with bone length or cortical bone responses (data not shown), suggesting that these bone parameter effects were not impacted by beneficial changes to the gut barrier.

## Discussion

Glucocorticoid‐induced osteoporosis is a devastating side effect of prolonged oral glucocorticoid use. Anti‐osteoporotic medications can be used successfully, but patient adherence (especially when young) can be difficult. The current study determined that probiotic treatment, specifically *Lactobacillus reuteri* ATCC 6475 (LR 6475), effectively prevented trabecular GIO in growing, oral GC‐treated male CD‐1 mice, but did not prevent decreases in spleen weight, bone length, cortical bone thickness and area, or cortical bone mineral content. The trabecular bone benefits were linked with LR 6475 enhancement of gut barrier function. This further supports the potential of including probiotic use as an adjunct to GC treatment to prevent GIO clinically with minimal side effects.

Several studies have established a link between gut and bone health.^(^ ^16^, ^17^, ^20^, ^22^, ^27^, ^31^, ^32^, ^33^, ^34^, ^35^, ^36^, ^37^ ^)^ There are several hypotheses, including a role for microbiota composition, microbial metabolites, immune cell activation, and barrier function. Our lab and others have identified gut barrier function as integral in both the pathogenesis and treatment of osteoporosis in several models, including a subcutaneous pellet model of GIO.^(^ ^16^, ^19^, ^27^, ^28^, ^38^, ^39^ ^)^ The link has been supported by correlations between gut permeability (serum levels of endotoxin and/or oral FITC‐dextran) and trabecular bone volume fraction. Importantly, an intestinal barrier enhancer prevents bone loss in conditions such as post‐ABX, GIO, and bacterial challenge, demonstrating the link between gut barrier and bone health.^(^ ^16^, ^17^, ^40^ ^)^ Several preclinical studies have investigated gut barrier function in the presence of systemic GC therapy and demonstrated impaired function.^(^ ^41^ ^)^ Our past results show that treatment with probiotic LR 6475 or a barrier enhancer (MDY) in adult, male C57BL/6J mice can effectively prevent the impaired barrier function observed with systemic, subcutaneous GC therapy and subsequently prevent the occurrence of GIO.^(^ ^16^ ^)^ However, this has not been tested in a model with oral GC delivery, where there is direct physical interaction between the GC, microbiota, gut barrier, and probiotic. In this study, we demonstrate that oral treatment with LR 6475 completely prevents the rise in serum endotoxin levels (measure of gut barrier function) in the GC‐treated groups. This is a significant finding as we have now demonstrated the beneficial effect of LR 6475 on barrier function (and trabecular bone volume) in two different models (subcutaneous GC pellet and oral GC) and mouse strains (C57BL/6J and CD‐1). VSL#3 did not have a significant effect, indicating that probiotic effects on the gut barrier and bone volume in this model may be probiotic specific. Further studies are needed to understand the mechanism by which LR 6475 improves gut barrier function under GC conditions and how this relates to prevention of GIO in this model.

One way to specifically enhance gut barrier function is through probiotic treatment. Additionally, and of major importance to this study, there are few, if any, side effects associated with their use. Previous studies by our lab and others have shown bone health benefits of probiotic use for menopause‐induced osteoporosis,^(^ ^22^, ^27^ ^)^ post‐antibiotic (ABX) dysbiosis‐induced bone loss,^(^ ^17^ ^)^ and bone loss associated with a variety of diseases, including type 1 diabetes,^(^ ^24^ ^)^ colitis,^(^ ^42^ ^)^ and obesity,^(^ ^28^ ^)^ among others. Our lab has also demonstrated that LR 6475 is particularly efficacious compared with *Lactobacillus rhamanosus* GG (LGG) in preventing post‐ABX bone loss as well as GIO in a subcutaneous GC pellet model,^(^ ^16^ ^)^ further supporting the idea that certain probiotics are preferentially efficacious in preventing bone loss in different disease models. Multiple probiotics have been tested in different labs and with varying levels of success. For example, Li and colleagues conducted an interesting study examining the effectiveness of several probiotic strains, including LGG and VSL#3 in preventing sex steroid deficiency–induced bone loss and found that LGG and VSL#3 both prevented femur and vertebral trabecular bone loss as well as prevented gut barrier dysfunction in C57BL/6J mice.^(^ ^27^ ^)^ VSL#3 was also shown to prevent high fat diet–induced bone loss and gut barrier dysfunction in C57BL/6J mice.^(^ ^28^ ^)^ Although others have shown prevention of bone loss and gut barrier impairments using VSL#3 in other models, we demonstrate here that VSL#3 is ineffective at preventing oral GC‐induced bone loss and barrier dysfunction. However, future studies testing its efficacy in other models such as the subcutaneous GC model are warranted.

A previous study examined overall (total body) bone mineral density and bone area via dual‐energy X‐ray absorptiometry (DXA) in the oral GC CD‐1 mouse model but did not investigate specific microarchitectural measures.^(^ ^30^ ^)^ In this study, we show that 75 μg/mL CS (~9 mg/kg/d) causes a significant reduction in both distal femur metaphyseal and vertebral trabecular bone volume, mineral content, and density compared with vehicle‐treated controls. Most importantly, oral treatment with LR 6475 was the only probiotic treatment to completely prevent bone loss at both sites (similar to our findings in other models). In contrast, treatment with VSL#3 did not prevent trabecular bone loss in the femur or vertebrae (Figs. 2 and 3). VSL#3 has not previously been investigated in GIO prevention. These results suggest that the prevention of GIO in this model may be probiotic specific (ie, only certain probiotics are protective), and future studies are warranted to better understand the probiotic‐specific effects found on bone volume.

As mentioned above, children can be placed on chronic GC therapy for a number of different health conditions, and it has been well documented that these children may experience reduced bone growth.^(^ ^43^ ^)^ Chronic GC therapy not only has negative effects on bone cells themselves (ie, osteoblast/osteocyte apoptosis), but also suppresses the hypothalamic–pituitary axis, resulting in a reduction in growth hormone production and subsequent reduction of IGF‐1 in the liver, both of which are integral to bone growth and development.^(^ ^43^ ^)^ In our study, we see a suppression in bone growth witnessed by a reduction in femur length in all GC‐treated groups. Neither probiotic was able to prevent this suppression. Future studies are needed to better understand potential mechanisms/approaches to prevent growth suppression in this model.

One reason we wanted to test the effect of LR 6475 in the oral GC model is to examine the potential for its use in human GIO. LR 6475 was shown to reduce bone loss in elderly women in a randomized, placebo‐controlled, double‐blind clinical trial.^(^ ^29^ ^)^ Women with a BMD *T*‐score between −1 and −2.5 at the spine or total hip were given placebo or 1 × 10^10^ CFU/d LR 6475 for 12 months. BMD and bone microstructure were assessed by quantitative computed tomography and DXA at baseline and after 12 months of treatment. Results revealed that bone loss was reduced via total volumetric BMD in the LR 6475 group. Although results did not reach statistical significance because of small sample size, this study laid the groundwork for future studies investigating LR 6475 as a clinically effective therapeutic option to prevent bone loss and demonstrates the translational promise of LR 6475 from preclinical models into the clinic.

Chronic GC use is known to reduce bone strength as assessed via structural properties of cortical bone.^(^ ^6^ ^)^ In our study, we demonstrate that there is a reduction in cortical thickness, cortical area, bone mineral content, and bone area after 4 weeks of GC treatment and that neither probiotic treatment was able to prevent this loss (Table 2). Our findings of reduced bone mineral content and bone area with GC treatment confirms the findings of the previous study using this model.^(^ ^30^ ^)^ Concurrently, we see a significant reduction in ultimate load, fail load, and stiffness in the GC‐treated groups and both probiotic treatments did not prevent this reduction (Table 3, Fig. 4). These results indicate that GC use in this model impairs structural properties of the femur cortical bone and that probiotic use is unable to mitigate this. It may be that the duration of treatment is not long enough to confer a benefit, and a longer treatment period is needed for benefits to cortical bone to be observed. Other studies examining the effect of probiotics on cortical bone in other models have found that single *Lactobacillus* strains or mixtures of *Lactobacillus* strains prevent changes in several measures, including cross‐sectional perimeter, cortical area, cortical thickness, and cortical bone mineral content resulting from estrogen deficiency in the menopausal osteoporosis model.^(^ ^23^, ^44^, ^45^ ^)^ Our lab has previously shown benefits of LR 6475 on cortical bone measures in mice in both a type 1 diabetes–induced model of bone loss^(^ ^24^ ^)^ and under inflammatory settings.^(^ ^26^ ^)^ Behera and colleagues show that biomechanical properties of the femur can be restored with VSL#3 in a high fat diet–induced model of bone loss.^(^ ^28^ ^)^ In our previous study examining the effect of LR 6475 in subcutaneous GC‐induced bone loss, we did not see changes in biomechanical properties or cortical bone with GC or probiotic treatment.^(^ ^16^ ^)^ It is unclear why there are different results on cortical bone and biomechanical properties between models and probiotic strain used. Future studies with longer time points are needed to completely understand the potential effects of probiotic treatment in mouse cortical bone and the overall effect on biomechanical and structural properties.

GCs are used therapeutically for their immunosuppressive role through a variety of mechanisms. One such mechanism is that GCs cause marked apoptosis of T and B cells in the spleen (the likely cause of decrease in spleen weight).^(^ ^46^ ^)^ In our study, we see significant reductions in spleen weight in all GC‐treated groups (with or without probiotics) after 4 weeks of treatment. This suggests that the LR 6475 does not affect the immunosuppressive effects of GCs but selectively inhibits the unwanted side effects of GCs on bone health. This is critical for the use of GC therapeutics for inflammatory diseases where the immunosuppressive effects are important to maintain.

Taken together, our study is the first to demonstrate that oral supplementation of probiotic LR 6475 is an effective method to prevent trabecular bone loss and GC‐induced gut barrier dysfunction in a clinically relevant, oral model of GIO in male, outbred CD‐1 mice. Future studies are needed to better understand the mechanisms behind which LR 6475 improves gut barrier function and prevents GIO.

## Author Contributions

**Nicholas John Chargo:** Data curation; formal analysis; writing – original draft; writing – review and editing. **Jonathan Daniel Schepper:** Conceptualization; data curation; formal analysis; methodology; project administration; writing – review and editing. **Naiomy Rios‐Arce:** Conceptualization; data curation; formal analysis; methodology; project administration; writing – review and editing. **Ho Jun Kang:** Data curation; formal analysis; methodology; writing – review and editing. **Joseph D. Gardinier:** Data curation; formal analysis; methodology; writing – review and editing. **Narayanan Parameswaran:** Conceptualization; funding acquisition; methodology; supervision; writing – original draft; writing – review and editing. **Laura R McCabe:** Conceptualization; funding acquisition; methodology; supervision; writing – original draft; writing – review and editing.

## Disclosures

LRM is a coinventor on patents related to LR 6475 and bone health. All other authors have no disclosures.

### Peer Review

The peer review history for this article is available at https://www.webofscience.com/api/gateway/wos/peer‐review/10.1002/jbm4.10805.

## Data Availability

Data available upon request to corresponding authors.
